# Concurrent Acute Monoblastic Leukemia and Multiple Myeloma in a 66-Year-Old Chemotherapy-Naive Woman

**DOI:** 10.14740/wjon722w

**Published:** 2014-05-06

**Authors:** Srujitha Murukutla, Swaty Arora, Vijaya Raj Bhatt, Shiksha Kedia, Muhammad Popalzai, Meekoo Dhar

**Affiliations:** a Department of Medicine, Staten Island University Hospital, 475 Seaview Avenue, Staten Island, New York 10305, USA; b Sanford R Nalitt Institute for Cancer and Blood Related Diseases, Department of Medicine, Division of Hematology and Oncology, Staten Island University Hospital, 256 Mason Avenue, Staten Island, New York 10305, USA

**Keywords:** Acute myeloid leukemia, Multiple myeloma, Chemotherapy

## Abstract

Concurrent acute myeloid leukemia (AML) and multiple myeloma (MM) is rare, more so in chemotherapy-naive patients. Concurrent occurrence of these two malignancies portends poor prognosis. Although anthracycline-based AML regimen, allogeneic hematopoietic stem cell transplantation, tipifarnib and bortezomib have shown promising results in small number of patients, there is a lack of established therapy. We describe a case of concurrent AML and MM in a 66-year-old woman and review previously published literature.

## Introduction

Concurrent acute myeloid leukemia (AML) and multiple myeloma (MM) is rare and presents unique therapeutic challenges. Here, we describe a case of concurrent AML and MM and review previously published literature.

## Case Report

A 66-year-old woman, complaining of fatigue, loss of appetite and 25-pound weight loss for 8 weeks, was seen in the ambulatory clinic at a different institution after detection of abnormalities in blood work. Her medical history was significant for 80 pack-year smoking, chronic obstructive pulmonary disease, hypercholesterolemia, hypertension, coronary artery disease, coronary stent placement, tonsillectomy and appendectomy. The patient had a good performance status. Family history was significant for breast cancer in her mother and esophageal cancer in her father. Her medications included isosorbide mononitrate, aspirin, simvastatin, metoprolol, inhalational ipratropium, albuterol and fluticasone.

Physical examination revealed temperature of 96 °F, blood pressure of 107/73 mmHg, pulse of 98/min and respiratory rate of 16/min. The patient was calm and oriented. She had pallor but no icterus, petechiae, or any other evidence of bleeding. Lymph nodes were not palpable. There was no bony tenderness. Abdominal examination did not reveal hepatosplenomegaly or any other masses. Rest of the physical examination was also unremarkable.

Laboratory tests revealed white count of 64,500/µL with 17% granulocyte, 11.9% monocyte and 6.2% lymphocyte, hemoglobin of 7.5 g/dL, platelet of 100,000/µL and serum lactate dehydrogenase level of 306 IU/L. Peripheral blood smear showed 3% blasts, 73% monocytes with some immature forms, 10% neutrophils, 2% myelocytes, metamyelocytes and promyelocytes, and 4% lymphocytes. Glucose, electrolytes, coagulation profile, renal and liver function tests were within normal limits.

A bone marrow biopsy done at the other institution revealed hypercellular bone marrow with decreased bone marrow precursor cells and infiltration with plasma cells, which were kappa restricted. Flow cytometry of the bone marrow aspirate was significant for two abnormal populations: 1) myeloid cells (27% of non-erythroid cells) with immunophenotype suggestive of monocytic differentiation; 2) plasma cells (33% of total cells) positive for monoclonal IgG, kappa-restricted, positive for CD38, CD117 and CD56 consistent with MM. The patient was referred to our institution for further management.

Peripheral blood smear at our institution showed 12% blasts with Auer rods. A repeat bone marrow biopsy showed hypercellular bone marrow (70-80%) with 50-60% blasts, decreased megakaryocytes, maturing myeloid and erythroid precursor cells and scattered interstitial plasma cells. Flow cytometric analysis revealed two abnormal cell populations: 1) 50-60% monoblasts with intermediate CD45 (LCA) expression with following immunophenotype: CD11c+, CD13+ (dim), CD33+, CD64+, CD14+/- (subset), CD38+, CD4+, CD7+ (dim), CD56+ (variable), CD117- and CD34-; 2) 1-2% atypical plasma cell population: CD38+ (bright), CD138+, CD56+. Karyotyping revealed normal female (46, XX). Immunohistochemical studies revealed monoclonal kappa light chain restricted plasma cells. Fluorescent *in situ* hybridization was negative for any chromosomal aberrations. Serum protein electrophoresis revealed total protein of 6.5 g/dL with immunofixation positive for monoclonal free kappa light chain band. Serum free kappa and lambda levels were 2820 mg/L and 18.2 mg/L respectively, with serum free kappa/lamda ratio of 154.9. Urine protein electrophoresis revealed monoclonal protein level of 1,901 mg/24 h with immunofixation positive for monoclonal free kappa light chain band. She had serum IgG of 821 mg/dL, IgM of 76 mg/dL and IgA of 131 mg/dL. Thus, a diagnosis of concurrent AML and asymptomatic MM was established. Multiple gated acquisition study revealed left ventricular ejection fraction of 60% with normal ventricular wall motion. A skeletal survey was negative.

The patient was started on allopurinol and hydroxyurea, and the latter was subsequently stopped once chemotherapy was initiated. She was induced as an in-patient with 7-day low-dose cytarabine (100 mg/m^2^/day) and 3-day idarubicin (12 mg/m^2^/day).

Her hospitalization was complicated by *Clostridium difficile* diarrhea, neutropenic sepsis, and hospital acquired pneumonia secondary to *Stenotrophomonas maltophila* and vancomycin resistant *Enteroccocus faecium*. She was aggressively treated in intensive care unit but she developed multiorgan failure and died on hospital day 23.

## Discussion

AML in a patient with MM is very rare and often occurs after chemotherapy for MM [1, 2]. Concurrent AML and MM in chemotherapy-naive patients is extremely rare with only few cases reported in the literature (Table 1) [3-17]. Acute myeloblastic or myelo-monocytic leukemia are the most common AML sub-types encountered in this setting. Postulated mechanisms to explain the concurrent occurrence of AML and MM include disorder of multi-potent stem cell [8, 18], exposure to common risk factors [6], leukemic or myeloma cells stimulating proliferation of bone marrow cells with subsequent development of a second hematologic malignancy [7] or AML occurring co-incidentally while monoclonal gammopathy of undetermined significance is progressing to MM [3].

**Table 1:** Review of previous reported cases of concurrent AML and MM

Author	Age	Sex	Type	Paraprotein	Karyotype	Treatment	Survival
Kim [3]	51	M	Myelo blastic	Κ	Complex	Bortezomib; ara-C and idarubicin; ara-C and mitoxantrone, allo-SCT	Alive at 421 days
Shukla [4]	58	F	Myelo blastic	IgG/λ	NA	Anthracycline/ara-C	1 month
Attili [5]	57	M	Myelo blastic	IgG/K	46, XY/t(8;21)	NA	Sepsis on day 7
Luca [6]	77	M	Monocytic	IgG/λ	Monosomy 13, structural changes of Chr 1, 20q-	NA	One month
Raz [7]	68	M	Monoblastic	IgG/K	“No bizarre chromosomal changes”	Cyclophosphamide followed 2 months later by melphalan followed by vincristine, melphalan, cyclophoside, prednisone	2.5 years
Cleary [8]	64	M	Myelomonocytic	IgA/K	46 XY	Cytarabine and daunorubicin	4 months
Tursz [9]	77*	M	Myeloblastic	IgA	NA	NA	NA
	74†	M	Myeloblastic	IgG//λ	NA	Mercaptopurine	Few months
Rosner [10]	79	M	Myelomonocytic	NA	NA	Cyclophosphamide 150 mg/day and testosterone for 6 weeks, which was stopped then after. Methotrexate and prednisone once AML was diagnosed 5 months later	Died shortly after the diagnosis of AML
Taddeini [11]**	73	M	NA	IgG//λ	NA	Combination chemotherapy	About 14 weeks
Parker [12]**	79	M	NA	IgG	Normal	Cytarabine and daunorubicin; then after, 5 days of prednisone and melphalan since plasma cell increased in repeat bone marrow exam	About 12 weeks
Kastanas [13]**	70	M	NA	IgG	NA	NA	NA
Annino [14]**	74	M	Myelomonocytic	IgG/K	Hypodiploidy and marker chromosome	NA	NA
Vallantin [15]**	66	M	Myeloblastic	IgG/λ	NA	NA	NA
Parapia [16]**	79	M		IgG/λ	Normal	Cytarabine, daunorubicin and 6-thioguanine	About 6 weeks

*Patient had pancytopenia for 3 years prior to the diagnosis. †Patient had pancytopenia for 5 years prior to the diagnosis. **Cases described in Rosner and Grunwald [17]. K: kappa light chain; λ: lambda light chain; F: female; M: male; NA: not available.

Reactive plasmacytosis is common in AML patients with an incidence of up to 6% and can be associated with monoclonal paraproteins in the absence of other components of the diagnostic criteria of MM [19]. Therefore, it is important to rule out reactive plasmacytosis before making a diagnosis of concurrent AML and MM.

Given the rarity of the disease, there has been no established treatment and hence, prognosis remains extremely poor. Patients are often treated with therapy for AML since AML is more aggressive and anthracyclines are effective against MM as well. There are two reported cases of concurrent MM and AML in chemotherapy-naive patients, who had better outcomes compared to other patients. Raz et al describe a 68-year-old man who was diagnosed in 1978 with MM and AML without any “bizarre chromosomal changes” on cytogenetic studies. The patient was initially treated with cyclophosphamide with disappearance of monoblasts. The disease, however, recurred in 2 months when he was treated with melphalan without success and thereafter with combination chemotherapy consisting of vincristine, melphalan, cyclophosphamide and prednisone. This resulted in the disappearance of monoblasts as well as significant decline in the serum level of paraprotein. Six months later, unfortunately he developed end-organ damage related to MM and became unresponsive to chemotherapy. He died of septic shock and severe bleeding tendency 2.5 years from the time of initial diagnosis [7]. Kim et al describe a 51-year-old previously healthy man who was diagnosed with concurrent MM and AML with complex cytogenetics and immunoglobulin heavy chain rearrangement. The patient was initially treated with one-cycle of bortezomib and then with cytarabine (ara-C) and idarubicin, along with bortezomib. This was followed by re-induction with mitoxantrone and high-dose ara-C and mitoxantrone (HAM regimen) for induction failure. Because of incomplete response, the patient subsequently went on receiving myeloablative allogeneic hematopoietic stem cell transplantation (allo-SCT) after conditioning with busulfan and cyclophosphamide. The patient remains to be disease-free and well at 421 days post-SCT [3]. There has been some promising result in a preclinical study in which tipifarnib and bortezomib have been shown to be synergistic in MM and AML cell lines [20]. Although the usefulness of these therapies needs further evaluation, because of the rarity of the disease, it will require an international registry to be able to do so.

### Conclusion

Although very rare, AML and MM can present simultaneously even in chemotherapy-naive patients. Concurrent occurrence of these two malignancies portends poor prognosis. Anthracycline-based AML regimens are often used because AML is more aggressive and anthracyclines are effective against MM as well. Allogeneic stem cell transplantation as well as tipifarnib and bortezomib have shown promising result.
